# Pharmacist's Perspectives on Administering a COVID-19 Vaccine in Community Pharmacies in Four Balkan Countries

**DOI:** 10.3389/fpubh.2021.766146

**Published:** 2021-11-25

**Authors:** Adina Turcu-Stiolica, Maria Kamusheva, Maria Bogdan, Ivana Tadic, Klejda Harasani, Mihaela-Simona Subtirelu, Andreea-Daniela Meca, Sofia Šesto, Marina Odalović, Jasmina Arsić, Svetlana Stojkov, Emili Terzieva, Guenka Petrova

**Affiliations:** ^1^Department of Pharmacoeconomics, University of Medicine and Pharmacy of Craiova, Craiova, Romania; ^2^Department of Organization and Economics of Pharmacy, Medical University-Sofia, Sofia, Bulgaria; ^3^Department of Pharmacology, University of Medicine and Pharmacy of Craiova, Craiova, Romania; ^4^Department of Social Pharmacy and Pharmaceutical Legislation, Faculty of Pharmacy, University of Belgrade, Belgrade, Serbia; ^5^Department of Pharmacy, Faculty of Medicine, University of Medicine of Tirana, Tirana, Albania; ^6^Department of Social Pharmacy, Faculty of Pharmacy, University Business Academy in Novi Sad, Municipio de Novi Sad, Serbia; ^7^Department of Biomedical Sciences, College of Vocational Studies for the Education of Preschool Teachers and Sports Trainers in Subotica, Subotica, Serbia

**Keywords:** COVID-19, vaccination, training, pharmacists, community pharmacy, Balkan countries

## Abstract

Community pharmacists expanded their roles and engaged in vaccination services in many countries around the world, but not in Balkan countries. This research aimed to assess the perceptions of pharmacists on involvement in the coronavirus disease (COVID-19) vaccine administration in four Balkan countries (Albania, Bulgaria, Romania, and Serbia). A cross-sectional survey was conducted using an online questionnaire that was distributed to community pharmacists across these countries between February and March 2021. A total of 636 community pharmacists were included in the analysis of the survey. The willingness to administer vaccines for COVID-19 (or other vaccines well established in the practice, like a flu vaccine) in community pharmacies is significantly different among the countries: the pharmacists from Albania were more willing to administer vaccines. The factors associated with the eagerness to vaccinate are almost the same among the countries: the lack of training in the faculty classes and the lack of a special place where to administer vaccines. Additional significant factors were found in Bulgaria (pharmacists from independent pharmacies wanted more than the pharmacists working in chain pharmacies to administer vaccines) and in Serbia (male pharmacists agreed more with administering vaccines than female pharmacists). Further national reforms are needed for adopting the expanding role of community pharmacists.

## Introduction

After declaring the new coronavirus disease (COVID-19) as a pandemic on March 11, 2020, WHO requested a collaboration among different countries and healthcare workers even though the global medical system faced multiple challenging novel responsibilities (1). Pharmacists have been involved worldwide in public health during this past year's pandemic through point-of-care testing services and by ensuring medication counseling, access, and optimization (2, 3). They have received greater authority from several governments all around the world to aid overcrowded hospitals (4). Various global studies underlined the importance of community pharmacist role expansion and engagement in vaccination services (5–9), leading to a more valuable and premature public health response that is potentially obtained during a pandemic if community pharmacists expanded their roles and engaged in vaccination services (6, 10).

In all European countries, pharmacists are authorized to provide patient-centered care by medicine optimization and dispensing the prescribed antibiotics and antiviral drugs to prevent infectious threats (11). The role of community pharmacies in vaccination has exceptional success in countries like Argentina, the United States of America, Australia, France, Ireland, Italy, Norway, Poland, Portugal, Switzerland, and United Kingdom, suggesting that pharmacists can play a greater role in improving vaccination coverage (12, 13). The question is if community pharmacists could mitigate public health emergencies such as the COVID-19 pandemic by also ensuring vaccination service. However, to manage vaccination services and ensure occupational safety, community pharmacies require flexibility and clarity in establishing workflows and basic infrastructure (1, 2).

First of all, the involvement of community pharmacists in vaccination services during pandemics may present some advantages as follows. Community pharmacies may offer a low and convenient cost for vaccination, being already prepared for vaccination logistics with multiple strategies for injection/vaccine supply chain management (14–17). They are often conveniently located and do not usually require appointments for vaccination services. This can increase access to vaccines that are medically suggested (18). Pharmacists are also able to combat misinformation, clearly communicate, and assess patient understanding (19), therefore decreasing vaccine hesitancy through persistent education (4) as human skepticism, and hesitancy regarding this unique health intervention deters immunization campaigns (20).

Second, community pharmacists may contribute to the prevention of public health crisis due to role expansion (2, 10). A global survey was conducted last year in over 99 countries and territories by the International Pharmaceutical Federation (FIP) and found that pharmacy-based vaccination services were available in at least 36 countries and territories and were proposed or were in the process of implementation in 16 more countries (21, 22). Among the vaccines administered in pharmacies, the most common was the influenza vaccine, followed by hepatitis B, tetanus, diphtheria, measles, malaria, and shingles vaccine. In the European Union, vaccination administrated in pharmacies is common in 13 European countries, including Greece, Portugal, and Estonia (23).

And thirdly, community pharmacist's involvement in public health services may be increased and improved after following specific training (24). For example, in the United States, pharmacists are trained to administer the vaccines approved by Food and Drug Administration (FDA), based on the Centers for Disease Control and Prevention (CDC) guidelines (5), and they have already improved influenza vaccination rates (2, 4, 10). Accredited workshops regarding best practices in pharmacy-based immunization services were organized in Quebec (Canada) for pharmacists and proved effective in encouraging vaccination and in improving their competencies (20).

Community pharmacist's training may include not only the study of epidemiology, immunology, and proper vaccine delivery, but also the appropriate use of protective equipment, correct vaccine storage and handling, and constant monitorization of the eligible population (10). Although an infectious disease pandemic is still difficult to predict nowadays, wide planning programs and medical staff preparedness could lead to higher psychological resilience among pharmacists (1). Therefore, a solution to overrun the COVID-19 pandemic might be expanding pharmacist's roles as they represent frontline medical professionals, highly responsive to patient's needs (2).

Although in some Balkan countries vaccination in pharmacies is regulated by laws, this is not yet possible in practice due to the lack of implementing procedures. The primary aim of this research is to analyze the perception of community pharmacists from the four Balkan countries (Albania, Bulgaria, Romania, and Serbia) toward vaccination services and the willingness to administer COVID-19 vaccines and other infectious disease vaccines to patients. The second purpose of our research was to identify the common pathways among these selected South-East European countries toward enhancing pharmacist's contributions and roles in vaccination by gathering information about the barriers and benefits to COVID-19 vaccination for community pharmacists.

## Materials and Methods

### Study Design

A cross-sectional survey was conducted using an online, anonymously self-administrated questionnaire that was distributed to community pharmacists across Albania, Bulgaria, Romania, and Serbia between February and March 2021. As dissemination channels, we used pharmacist groups from social media and emails to our former students.

To calculate an appropriate representative sample from the targeted population, we established the margin of error at 5% with 95% confidence level. The number of Albanian community pharmacists is 2,744 (25), so the required sample size is 338. The number of Bulgarian pharmacists registered in the database of the Bulgarian Pharmaceutical Union is 6,605 (26), so the required sample size is 364. The number of Romanian pharmacists registered in the database of Romanian Pharmacists College is 18,093 (27), and the required sample size is 377. The number of Serbian licensed pharmacists is 7,147 (28), so the required sample size is 365.

Only complete surveys were included in the analysis.

### Survey Development

The survey was developed based on a deep literature review (29, 30) and the experience of the four countrie's coordinators (KH, MK, AT-S, and IT) who analyzed the structure, content validity, and applicability in the selected countries. The survey consisted of 19 items, including both close-ended questions with predefined answers and open-ended questions. The items were grouped into three sections: the first section was dedicated to the sociodemographic data (age, gender, marital status, specialty, years of experience working as a pharmacist, and community pharmacy characteristics), the second section assessed the perceptions about administering vaccines (Q1–Q8 close-ended questions), and the third section was about barriers and facilitators on vaccine administration in community pharmacies (Q9–Q10 open-ended questions).

A pilot test was conducted on 20 community pharmacists (5 in each country), who were not included in our sample, to assess the clarity, understandability, and relevance of the survey items. The principal investigators of the four countries (Albania, KH; Bulgaria, MK; Romania, AT-S; and Serbia, IT) discussed with the community pharmacists from their countries. Their feedback had helped for face content validity and to obtain the final version of the survey.

### Statistical Analysis

All statistical analyses were performed using the GraphPad Prism 9.2.0 software (GraphPad Software, San Diego, CA, USA). Descriptive data were represented by mean with SD, median with an interquartile range for continuous variables, and frequencies with percentages for categorical variables. The Mann–Whitney U and Kruskal–Wallis tests were used to test the differences between the data obtained in participating countries. Univariate and multivariate analyses were performed to find the factors influencing the perception of administering vaccines in community pharmacies, and the adjusted odds ratio (AOR) was calculated. The 95% CI for the odds ratio was assessed for every predictor. The Hosmer and Lemeshow test is used to assess how well the model fits with the data. The results with the value of *p* < 0.05 were considered as significant.

### Ethical Approval

The protocol of this study was based on an online survey to which community pharmacists from every country (Albania, Bulgaria, Romania, and Serbia) answered voluntary, in compliance with European ethical recommendations on the absolute confidentiality of personal data collected in the survey, as well as the anonymity and security of participants (Regulation (EU) 2016/679 on the protection of individuals regarding the processing of personal data). A collaboration agreement was signed and approved by the Ethics Commission of the University of Medicine and Pharmacy of Craiova (No. 18/12.02.2021). All pharmacists provided electronic informed consent, starting with the first question of the survey.

## Results

A total of 636 community pharmacists from Albania (*n* = 109), Bulgaria (*n* = 168), Romania (*n* = 171), and Serbia (*n* = 188) were included in the analysis of the survey. Table 1 presents the sociodemographic characteristics of the respondents. The pharmacists from Albania were younger and less experienced than those from Bulgaria, Romania, or Serbia (age: Albania vs. Bulgaria, *p* < 0.0001, Albania vs. Romania, *p* < 0.0001, Albania vs. Serbia, *p* < 0.0001; experience working as pharmacist: Albania vs. Bulgaria, *p* = 0.0028, Albania vs. Romania, *p* = 0.0002, Albania vs. Serbia, *p* < 0.0001).

**Table 1:** Sociodemographic characteristics of pharmacists.

	**Albania**	**Bulgaria**	**Romania**	**Serbia**	***p*-value**
	**(*n =* 109)**	**(*n =* 168)**	**(*n =* 171)**	**(*n =* 188)**	
**Age (years)**					<0.0001
Mean (±SD)	29.6 (±6.3)	35 (±11.1)	33.61 (±6.8)	39.5 (±11.5)	
Median (IQR)	27 (25–33)	30 (26–42.8)	32 (28–39)	39 (29–48)	
**Gender (female, #)**	95 (87.2%)	140 (83.3%)	157 (91.8%)	159 (84.6%)	0.083
**Marital status (#)**					<0.0001
Married	47 (43.1%)	92 (54.8%)	92 (53.8%)	104 (55.3%)	
Not married	61 (56%)	67 (39.9%)	71 (41.5%)	69 (36.7%)	
Divorced	1 (0.9%)	9 (5.4%)	8 (4.7%)	15 (8.1%)	
**Specialty (yes, #)**	83 (76.1%)	21 (12.5%)	63 (36.8%)	54 (28.7%)	<0.0001
**Experience working as a pharmacist (years)**	5.8 (±5.7)	10.3 (±10.8)	7.9 (±6.2)	12 (±10.3)	<0.0001
Mean (±SD)	3 (1.25–9)	6 (2–15)	6 (3–11)	10 (3–20)	
Median (IQR)					
**Location of community pharmacy (#)**					0.004
Urban	105 (96.3%)	161 (95.8%)	157 (91.8%)	163 (86.7%)	
Rural	4 (3.7%)	7 (4.2%)	14 (8.2%)	25 (13.3%)	
**Is the community pharmacy you work for next to a hospital? (“yes”, #)**	38 (34.9%)	66 (39.3%)	43 (25.1%)	96 (51.1%)	<0.0001
**Type of a community pharmacy you work for (chain, #)**	24 (22%)	86 (51.2%)	107 (62.6%)	154 (81.9%)	<0.0001

*SD, standard deviation; IQR, interquartile range*.

*^#^ Data are presented as frequency (percentage)*.

The Kruskal–Wallis test was conducted to compare the willingness to administer vaccines in the community pharmacies of different countries (Albania, Bulgaria, Romania, and Serbia), and a significant difference was found among them, as shown in Table 2. The pharmacists from Albania were significantly more willing to administer vaccines in community pharmacies than those from Bulgaria, Romania, and Serbia (Q2 item: Albania vs. Bulgaria, *p* < 0.0001, Albania vs. Romania, *p* < 0.0001, Albania vs. Serbia, *p* < 0.0001). The same difference between the pharmacist's beliefs was maintained if the vaccines are well established in the practice (like a flu vaccine, Q3 item) or new (like COVID-19 vaccine, Q4 item). The beliefs of pharmacists about how the vaccination service should be paid or free of charge are also significantly different, Romanian pharmacists agreed more than from other countries that this pharmaceutical service must be covered by national health insurance funds (Q5–Q7 items). Most of the pharmacists agreed upon the national health insurance funds should pay the vaccination service provided by the pharmacists in a community pharmacy, regardless of the country (56, 57, 71, and 69% of the pharmacists from Albania, Bulgaria, Romania, and Serbia, respectively).

**Table 2:** The survey results.

**Items [frequency of “yes” answer (percentage)]**	**Albania**	**Bulgaria**	**Romania**	**Serbia**	***p*-value**
	**(*n =* 109)**	**(*n =* 168)**	**(*n =* 171)**	**(*n =* 188)**	
Q1	104 (95.4%)	112 (66.7%)	98 (57.3%)	112 (59.6%)	<0.0001
Q2	99 (90.8%)	76 (45.2%)	91 (53.2%)	102 (54.3%)	<0.0001
Q3	101 (92.7%)	89 (53%)	92 (53.8%)	106 (56.4%)	<0.0001
Q4	81 (74.3%)	56 (33.3%)	65 (38%)	76 (10.4%)	<0.0001
Q5	61 (56%)	28 (16.7%)	35 (20.5%)	57 (30.0%)	<0.0001
Q6	69 (63.3%)	104 (61.9%)	136 (79.5%)	118 (62.8%)	0.001
Q7					<0.0001
National health insurance	61 (56%)	96 (57.1%)	122 (71.3%)	129 (68.6%)	
Patient	11 (10.1%)	32 (19%)	17 (9.9%)	23 (12.2%)	
No payment	23 (21.1%)	37 (22%)	10 (5.8%)	32 (17%)	
Others	14 (12.8%)	3 (1.9%)	22 (12.9%)	4 (2.1%)	
Q8	89 (81.7%)	73 (43.5%)	84 (49.1%)	105 (55.9%)	<0.0001

*Q1: “Do you agree that pharmacy students must be trained to administer vaccines in a community pharmacy and administering vaccines should be part of the curricula taught to pharmacy students?”*;

*Q2: “Do you agree that pharmacists could administer vaccines (in general) in a community pharmacy”*.

*Q3: “Do you agree that pharmacists could administer well-established vaccines (safe, with rare adverse drugs reactions, as, for example, the flu vaccine) in a community pharmacy?”*

*Q4: “Do you agree that pharmacists could administer new vaccines (as Coronavirus disease (COVID-19) vaccine) in a community pharmacy?”*

*Q5: “Do you agree that pharmacists would provide the vaccination service for free because, after all, they have benefited from the sales?”*

*Q6: “Do you think the vaccination service provided by the pharmacists should be paid?”*

*Q7: “Who should pay for the vaccination service provided in pharmacies?”*

*Q8: “Do you think the community pharmacy where you work could arrange a special space for administration of the vaccines by pharmacists.”*

In accordance with the differences found among the four countries (as presented in Table 2), the binary logistic regression analyses were adjusted for each participating country. According to the binary logistic regression, the factors associated with the willingness to administer vaccination in community pharmacies are almost the same, as given in Tables 3–6.

**Table 3:** Logistic regression analysis of significant factors associated with the willingness of administering vaccination in the community pharmacies from Albania.

	**COR**	**95% CI**	***p-*value**	**AOR**	**95% CI**	***p-*value**
Age	1.04	0.92–1.17	0.55			
Gender (female)	0.74	0.09–6.29	0.78			
Marital status (married)	0.47	0.13–1.78	0.27			
Specialty (no)	0.43	0.11–1.66	0.22			
Experience	1.02	0.9–1.15	0.75			
Environmental (rural)	0.28	0.03–2.99	0.29			
Near hospital (no)	0.44	0.09–2.17	0.31			
Pharmacy type (chain)	2.72	0.33–22.65	0.35			
Q1 (no)	0.02	0.001–0.16	<0.0001	0.03	0.001–0.82	0.038
Q8 (no)	0.014	0.002–0.12	<0.0001	0.02	0.002–0.001	0.001

*COR, crude odds ratio; AOR, adjusted odds ratio*.

*Cox & Snell R^2^ = 0.274; Nagelkerke R^2^ = 0.59; accuracy = 94.5%; Hosmer and Lemeshow test, p = 0.131*.

**Table 4:** Logistic regression analysis of significant factors associated with the willingness of administering vaccination in the community pharmacies from Bulgaria.

	**COR**	**95% CI**	***p-*value**	**AOR**	**95% CI**	***p-*value**
Age	1.02	0.99–1.05	0.13			
Gender (female)	0.67	0.29–1.51	0.33			
Marital status (married)	3.15	0.73–13.72	0.13			
Specialty (no)	0.37	0.14–0.96	0.04	0.41	0.13–1.31	0.133
Experience	1.02	0.99–1.05	0.12			
Environmental (rural)	7.8	0.92–66.28	0.06	3.32	0.3–36.14	0.325
Near hospital (no)	0.99	0.53–1.84	0.96			
Pharmacy type (chain)	0.38	0.2–0.71	0.002	0.37	0.17–0.81	0.014
Q1 (no)	0	0	0.99			
Q8 (no)	0.08	0.04–0.17	<0.0001	0.08	0.04–0.17	<0.0001

*COR, crude odds ratio; AOR, adjusted odds ratio*.

*Cox & Snell R^2^ = 0.329; Nagelkerke R^2^ = 0.44; accuracy = 88%; Hosmer and Lemeshow test, p = 0.316*.

**Table 5:** Logistic regression analysis of significant factors associated with the willingness of administering vaccination in the community pharmacies from Romania.

	**COR**	**95% CI**	***p-*value**	**AOR**	**95% CI**	***p-*value**
Age	1	0.96–1.05	0.99			
Gender (female)	0.84	0.28–2.54	0.76			
Marital status (married)	0.24	0.05–1.29	0.09			
Specialty (no)	0.42	0.22–0.79	0.008	0.66	0.22–1.98	0.46
Experience	1.05	0.99–1.1	0.07			
Environmental (rural)	0.64	0.21–1.92	0.42			
Near hospital (no)	0.45	0.22–0.94	0.03	0.62	0.15–2.49	0.49
Pharmacy type (chain)	0.67	0.36–1.26	0.21			
Q1 (no)	0.01	0.003–0.03	<0.0001	0.011	0.003–0.04	<0.0001
Q8 (no)	0.085	0.04–0.17	<0.0001	0.094	0.029–0.31	<0.0001

*COR, crude odds ratio; AOR, adjusted odds ratio*.

*Cox & Snell R^2^ = 0.576; Nagelkerke R^2^ = 0.77; accuracy = 89.5%; Hosmer and Lemeshow test, p = 0.236*.

**Table 6:** Logistic regression analysis of significant factors associated with the willingness of administering vaccination in the community pharmacies from Serbia.

	**COR**	**95% CI**	***p-*value**	**AOR**	**95% CI**	***p-*value**
Age	0.99	0.96–1.02	0.42			
Gender (female)	0.41	0.17–0.97	0.04	0.145	0.02–1.004	0.05
Marital status (married)	3.15	0.8–12.42	0.1			
Specialty (no)	0.78	0.41–1.47	0.44			
Experience	0.99	0.96–1.02	0.44			
Environmental (rural)	2.98	1.13–7.84	0.03	1.003	0.24–4.15	0.99
Near hospital (no)	0.89	0.5–1.58	0.68			
Pharmacy type (chain)	0.52	0.24–1.13	0.1			
Q1 (no)	0.005	0.001–0.02	<0.0001	0.004	0.001–0.02	<0.0001
Q8 (no)	0.23	0.12–0.42	<0.0001	0.38	0.13–1.13	0.08

*COR, crude odds ratio; AOR, adjusted odds ratio*.

*Cox & Snell R^2^ = 0.595; Nagelkerke R^2^ = 0.78; accuracy = 92%; Hosmer and Lemeshow test, p = 0.219*.

Table 3 summarizes the results of the univariate and multivariate analysis to describe the factors associated with the willingness to administrate vaccines in community pharmacies in Albania. Pharmacist's willingness to administer vaccination is related only with the lack of training in the faculty classes and the lack of a special place where to administer vaccines into a community pharmacy.

The community pharmacists from independent Bulgarian pharmacies wanted more than the community pharmacists from chain pharmacies to administer vaccines. Also, the specialist pharmacists agreed more than non-specialist pharmacists to administer vaccines. The most significant factor influencing the willingness to administer vaccines in Bulgaria is the lack of space to do it.

The community pharmacists from Romania considered that it is important for a pharmacy to be located near a hospital. Like Bulgarian pharmacists, the specialist pharmacists from Romania were willing more than non-specialist pharmacists to administer vaccines, but not significant in the multivariate analyses. Pharmacist's willingness to administer vaccination is related only with the lack of training in the faculty classes and the lack of a special place where to administer vaccines into a community pharmacy.

In Serbia, male pharmacists agreed more with administering the vaccines than female pharmacists. The lack of training in the pharmacy faculty classes is the most important factor in the willingness to administer vaccines in the community pharmacies of Serbia.

**Figure 1:**
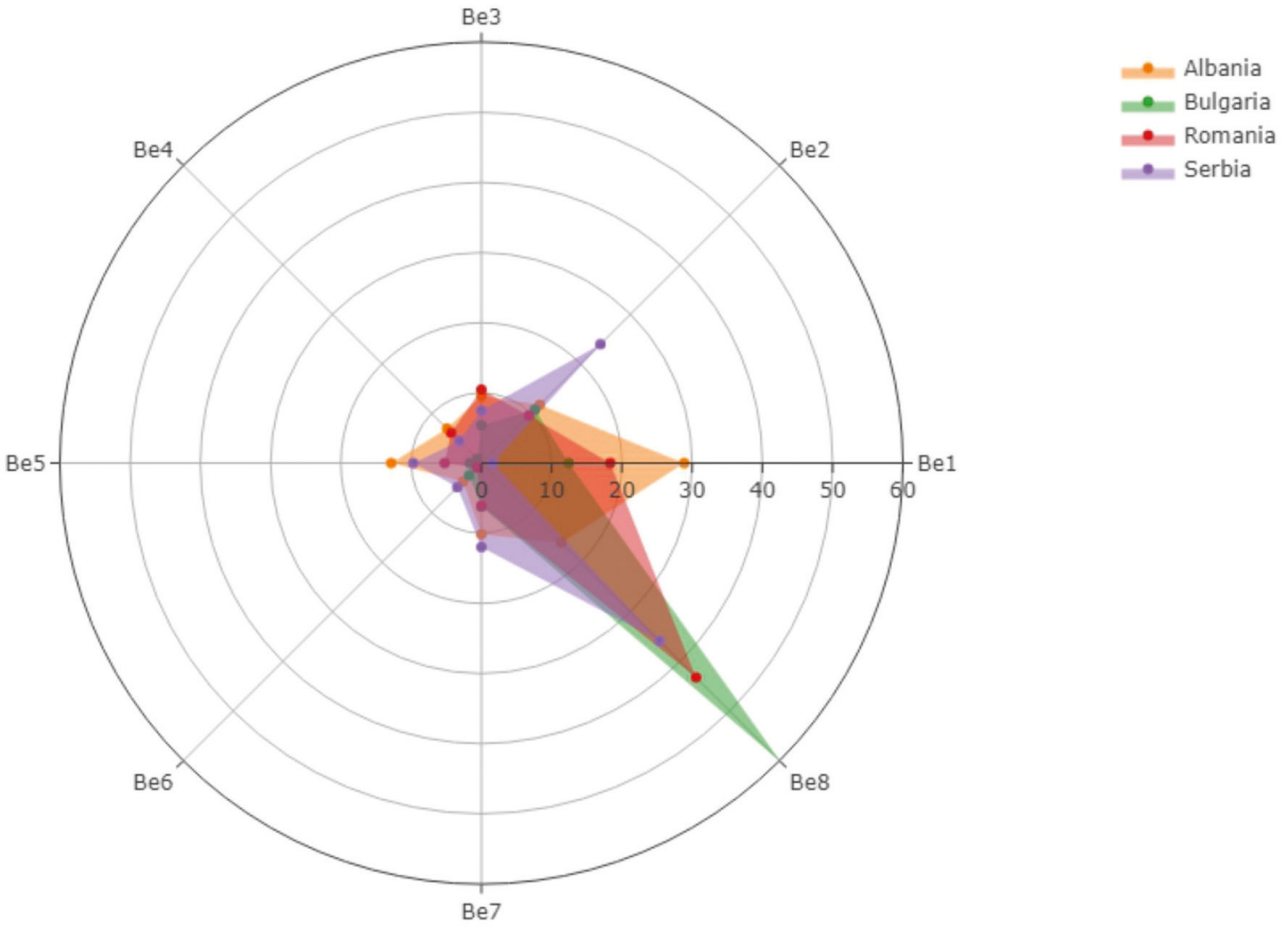
The benefits that would be offered through vaccination services provided in community pharmacies.

The answers offered by pharmacists regarding the benefits that would be offered through vaccination services provided in community pharmacies were summarized in Figure 1, and different opinions were collected from country to country. For example, the pharmacists from Albania considered time-saving for patients being a benefit of 28.87%, whereas the pharmacists from Serbia only have 1.65%.

**Figure 2:**
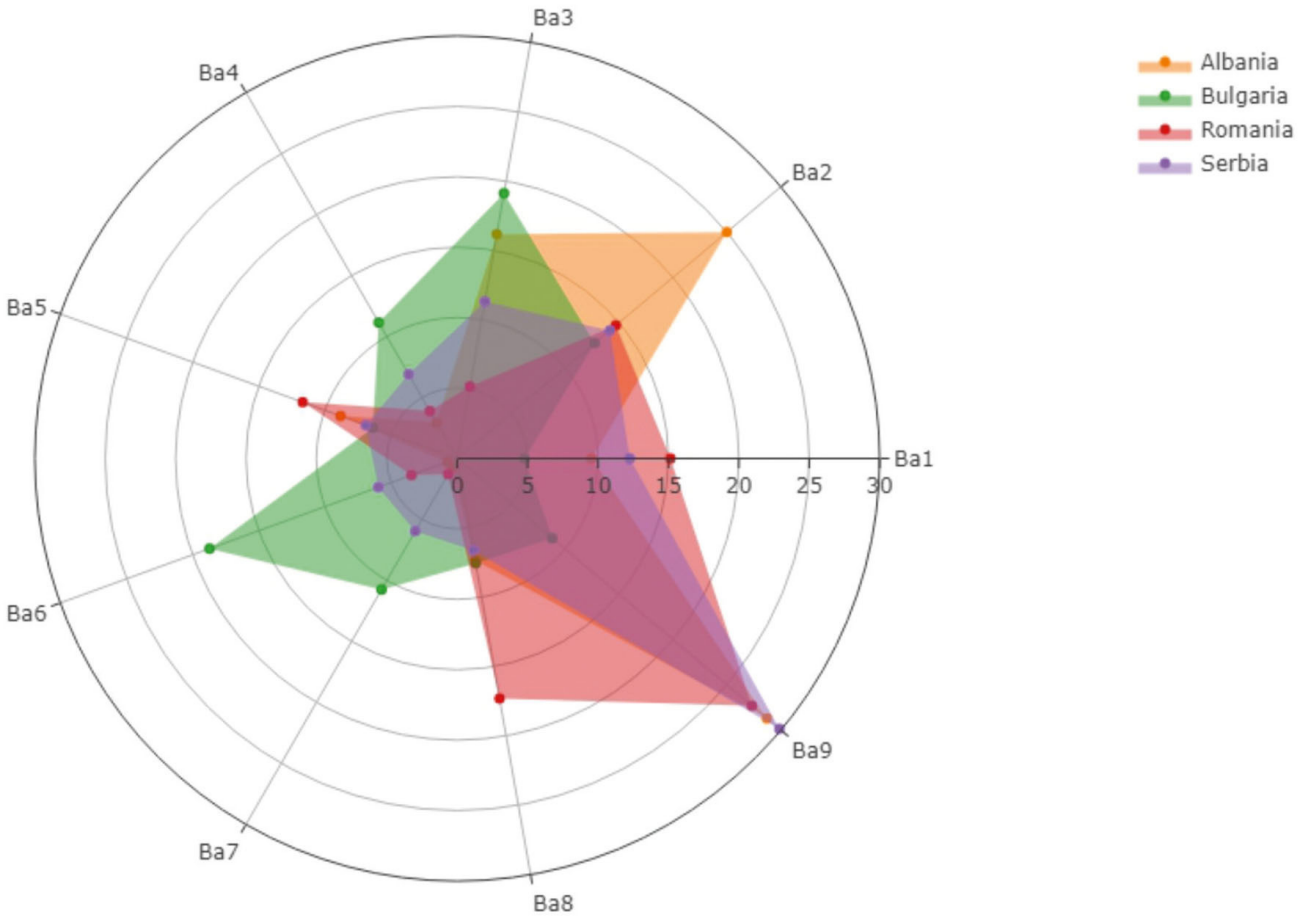
The barriers that must be overcome through vaccination services provided in community pharmacies.

The observed barriers to be overcome through vaccination services provided in community pharmacies are condensed and given in Figure 2. Different opinions could be remarked between the analyzed countries: for example, 0.73% from Albanian pharmacists and 18.72% from Bulgarian pharmacists considered increased workload for pharmacists being a barrier that must be overcome if vaccination services will be provided in community pharmacies.

## Discussion

Since the emergence of the severe acute respiratory syndrome coronavirus 2 (SARS-COV-2) global pandemic, more than 4 million deaths have been confirmed worldwide and 1.2 million deaths have been declared by WHO in Europe in the last year due to the pandemic (31), requesting coordination between existing health services to faster vaccinate the population in large scale (32–35). European Medicines Agency (EMA) and various national regulatory authorities have approved the clinical use of some vaccines following preclinical trials with promising results on decreasing hospitalization and COVID-19 mortality rates (32, 33, 36). There is an urgent need to reach at least 67% vaccination rates in the shortest possible time to reduce disease severity (32, 33). In this direction, our study is the first research that examined the perceptions of pharmacists in Balkan countries about administering COVID-19 vaccines and other well-established vaccines.

Similar to other countries such as Belgium, Croatia, Germany, Czech Republic, The Netherlands, pharmacists from South-East European countries (Albania, Bulgaria, Romania, and Serbia) ensure proper vaccine storage, dispensing, stock management, and participate in vaccination campaigns as promoters, but are legally not allowed to administer vaccines (32, 37). Nevertheless, in other countries such as Jordan, pharmacists are not the ones who vaccinate, they can only prescribe over-the-counter medications. Despite an urgent need to extend the prescribing role during the COVID-19 pandemic, the opportunity for pharmacists to extend their prescribing authority has not emerged yet (8, 9, 38). On the contrary, community pharmacists in Italy got the legislative approval to administer vaccines such as AstraZeneca and Johnson & Johnson after completing an obligatory training course and only in pharmacies with a specific infrastructure containing areas of reception, administration, and saving patient's data (39). Until the COVID-19 pandemic burst, vaccines were administered in pharmacies in Italy by nursing staff employed especially for vaccine administration and not by pharmacists (34). Even more, in Switzerland, community pharmacists are allowed to vaccinate against influenza, tick-borne encephalitis, or any other disease (including COVID-19 infection) in the different cantons of the country, after obtaining a specialized training certificate. Since 2018, pharmacies in Norway have increased the vaccination coverage rate by administering vaccines to patients older than 12 years entitled to a prescription from a physician. In France, as well as in Portugal, patients without a history of an allergic reaction can independently decide where to get the flu vaccine and can be vaccinated by pharmacists who complete a special training program (34).

Our survey focused upon the pharmacist's perceptions regarding the barriers that pharmacists believe must be overcome for the administration of vaccines and the benefits that would be offered through vaccination services provided in the community pharmacies from the European countries included in our study. More than 67, 68, and 98% of the pharmacists from Romania, Albania, and Bulgaria, who were included in the analysis, offered different thoughts or solutions about the implementation of vaccination services in community pharmacies. Although some of the participants are eager to evolve professionally and need to be seen as a credible healthcare human resource, others are reluctant toward administering vaccines. The lack of training and the lack of a special space for this activity are the most important barriers, similar to other studies (40–42). Therefore, assessing various practices and vaccine delivery possibilities as part of multifaceted pharmaceutical care remains challenging (34, 43, 44).

A Romanian pharmacist considers that vaccination services provided within community pharmacies could “increase the trust of general population in pharmaceutical services, could determine patients to seek more often the professional advice of a pharmacist, and could consequently, gain patients” support for this profession, further leading to legislative changes in its approval. The pharmacists from Albania recall the importance and involvement of pharmacists as first-line professional medical personnel not only during pandemic times but all the time as “the patient is always directed to the pharmacy first and it would be meaningful to administer the vaccine there.” Another Romanian pharmacist concludes that “the only advantage, especially in the pandemic situation, would be the large-scale immunization of the population” as a mitigation strategy.

Most of the Romanian surveyed pharmacists believe that “the legislation on the compartmentalization of the pharmacy must be changed, including a vaccination space” and one pharmacist suggests that “at least a few pharmacies in each city could represent a compromising beginning” in facilitating vaccination services within community pharmacies. Bulgarian pharmacists also think “it would be good for a patient to be able to get vaccines, without being mandatory for all pharmacies,” but after ensuring proper training and education for pharmacists within faculties of pharmacy. Some of the pharmacists proposed to attend special courses for both vaccination and first aid in case of the appearance of vaccine side effects. Pharmacists might be in an impossible position to provide a fast and adequate first aid in case of an adverse reaction, including an anaphylactic shock, while administrating a vaccine, as a pharmacist from Bulgaria confirms. This could imply, as a Romanian pharmacist points out, a “stronger psychological barrier of the patient” and could lower even more their trust toward pharmacist's activity. Another study that included European pharmacist's opinions mentions their lack of training and knowledge of providing clinical procedures and lack of recognition of their expertise as barriers in their pharmaceutical care (39). Pharmacist's roles and preparedness have never been more attractive to take into consideration as part of the interdisciplinary care until now (42, 43). Paudyal et al. (39) also reported the need of some European pharmacists for training regarding the effective use of protective equipment. For example, in Croatia, a novel course about pharmaceutical vaccination services (“Flu-vaccination in Pharmacy Practice”) was introduced in 2021 in the obligatory curriculum of the Faculty of Pharmacy (39). Jacob et al. (44) stated that in the USA, according to a national survey prior to the use of COVID-19 vaccines, most pharmacists would receive (78%) and recommend (81%) a COVID-19 vaccine. In Portugal, every 5 years, pharmacists are obliged to participate in training regarding vaccine delivery (34). The curriculum of the master program at the University of Belgrade—Faculty Pharmacy is adapted to the new and pharmacy services to be implemented and therefore several courses cover the vaccination services. The guidelines for flu vaccination in community pharmacies are prepared by the Pharmaceutical Chamber of Serbia, and the legislative approval is waiting (32). Furthermore, the pharmacists from Bulgaria draw attention to their inability to track all diseases and conditions of patients imposing additional risks for both pharmacists and patients. Some pharmacists from Albania, Romania, and Serbia proposed the idea of “an online platform that could contain the medical history of each patient,” and all health professionals could have access to this platform. Paudyal et al. confirmed the lack of access for pharmacists to patient's clinical records (39). This could also lead to improving the relationship between pharmacists and physicians. Even more, a proper monitorization of a person's health by all health professionals could also reduce risks for pharmacists, as a pharmacist from Bulgaria mentioned: “Vaccination should be tailored to the person's health. Pharmacists do not have access to complete patient information and cannot judge whether a person's vaccination is appropriate for them. Many factors, such as chronic and autoimmune diseases, need to be considered. In addition, pharmacists do not have information on whether the patient has viral diseases such as AIDS and hepatitis, which poses a risk to the health of the pharmacist himself.” A participant from Albania proposed a collaboration between pharmacists and physicians during vaccination, which could ensure medical advice given to community pharmacists when needed but did not expand the answer with possibilities of idea implementation.

Collaboration with healthcare professionals is very important as some Romanian and Bulgarian pharmacists consider that pharmacists should not be confounded with physicians since they have not received vaccination training during faculties. In addition, some of them suggest that vaccination services provided among community pharmacies could increase patient's trust in pharmacist's activity and could meliorate the opinion of the general population about the status of the pharmacists. Dawoud et al. also described the pressure of pharmacists who constantly aim to gain patient's trust (43). One pharmacist even mentioned that a possible “conflict of interests” could appear but did not expand the response. One of the most submitted advantages of providing vaccination services among community pharmacies is represented by a “partial relief in physician's tasks” and consequently “lowering the burden of family doctors,” ensuring time economy for patients and medical staff, consequently with other research studies (42, 46). One of the specialists from Bulgaria believes that administering a vaccine with the help of the pharmacists could ensure “better distribution of people, lack of long queues in front of hospitals, less risk of infection and transmission of the disease, greater choice of the patient where to get the vaccine.” Paudyal et al. conducted a qualitative study among the pharmacists from 16 European countries to identify possible novel actions for pharmacists to provide clinical service during pandemic times and therefore to ensure the recognition of pharmacists as public healthcare providers (39). A pharmacist from Romania also noted that vaccination within community pharmacies could lead to larger vaccine accessibility of patients who would be provided prompt medical services “leading to an improvement in the quality of human life.” The pharmacists from all Balkan countries included in our study agree upon the general mentality of the population and the difficulty of the pharmacist's reposition as first-line health professionals, underlining the need to strengthen the relationship between pharmacists and patients. A generalized tendency of individuals to have superior trust in the doctor's advice has been noted by some pharmacists from different European countries included in the present analysis, consistent with other studies (43, 46, 47). Even more, several other studies confirmed the difficulty of patients to accept the advice provided by a pharmacist (39, 42, 43, 47).

A study conducted by Austin et al. interviewed several community pharmacists and reported that task-focus, a well-organized schedule, shorter shifts, and consistent teamwork rather than an individual increased their ability to cope with pressure and improved their professional practice quality (1). A Romanian pharmacist underlines their overwhelmed position in community pharmacies as they sometimes “feel as they are imposed to volunteer,” due to “multiple extra-specialized tasks, management of sales reports, general accounting activities” as another colleague from the same country mentions. Another Romanian pharmacist complains about “minimum staffing schemes” and underlines their necessity to fulfill “activities anyway far beyond the job description,” while a Bulgarian colleague believes that the possible advantages from vaccination services within community pharmacies “would be only for the doctors if another of their obligations was dropped.” Even more, a great number of Bulgarian pharmacists think that increasing community pharmacist's task will determine an “influx of people in the pharmacies and providing vaccines for them will complicate” even more “their other duties.”

Community pharmacists are already involved in verifying shelves medication stocks, ensuring an uninterrupted supply chain by keeping a constant connection with producers and deposits, and predicting sales through inventories (1, 2, 46, 48), or different interventions and services (49). European pharmacists have already been involved in disease control and prevention of infection in the past year and directly ensured healthcare directed toward patients as a response to the pandemic crisis (39, 42, 43, 46). In Croatia, pharmacists have also been involved in the production of hand sanitizers and online counseling using telemedicine devices (32). To prevent the spread of SARS-COV-2, pharmacists communicated with patients and provided proper explanations about the importance of social distancing and the correct use of protective equipment (39, 41, 43, 48). Most pharmacists from Albania claim the need for higher organizational preparedness, after the lack of educational vaccination programs, consistent with other studies (34, 50). Their colleagues from Bulgaria draw attention to the possibility of increased workload for pharmacists while relieving physician's burden. Moreover, the pharmacists from Romania address legislative barriers with a more commonly mentioned reluctance in comparison to their neighbors. A systematic review conducted by Burson et al. revealed that despite a higher rate of vaccine service acceptance from both pharmacists and patients, legislative and administrative barriers limit the effectiveness of vaccine administration within community pharmacies (50). Even though it did not represent the main focus of their study, Austin et al. underline that all the pharmacists agreed upon the regression of clinical services such as pharmacovigilance or vaccinations during the COVID-19 pandemic lockdown in Canada, possibly due to overloaded work and social distancing recommendations (1). Prescription checkups, management of cleaning protocols, educating patients, and combating fake news due to their communication skills, as well as monitoring medication inventories emphasize multitasking behavior of community pharmacists (1, 39). This behavior has been described, however, as dramatically stressful and can disrupt pharmacy practice (1, 51). Adjustments in the schedule of community pharmacies (32), increasing the number of human resources (43), and prioritizing pharmacovigilance services through interdisciplinary teamwork (41, 43, 50) could minimize fatigue and distress in personnel (39, 43).

Multi-professional care conducted by pharmacists could facilitate their interventions in maintaining vaccine availability, distribution, and storage (10, 43, 45). A study conducted by Gessler et al. underlined that the extension of operation hours could be necessary to manage large crowds and to increase pandemic vaccination rates (10). The pharmacists from all the South-East European countries included in our study claim the necessity “to allocate sufficient time for each patient willing to vaccinate” and to enlarge the pharmaceutical teams, otherwise, there would “be constant queues in front of the work establishments” as an individual from Bulgaria confirms and “other patients will have to wait and will get nervous” as his colleague from Albania appreciates. As a solution, a pharmacist from Bulgaria came with the proposal of booking some hours during the pharmacist's program for vaccination service. Moreover, multiple differences in the perceptions of the participants have been noted as some of them consider “already too many responsibilities” while others comment that “there are many cases when patients themselves have addressed us and asked us to offer such a service [vaccination].”

Pharmacist's role in vaccine logistics is a very important aspect of vaccination success in a community pharmacy, since vaccine supply chain, stock, and storage management have already been established through the pharmaceutical national laws (14–17). The majority of participants agreed on the benefit of avoidance of precarious conditions and temperature fluctuations during transport and improper storage of the vaccine by a patient until the moment of administration if the vaccine was administered in a community pharmacy, consistent with other studies (40–43). Even more, pharmacists also proposed postvaccination counseling services as a possible benefit of vaccination provided by them, confirmed appropriate by researchers and authorities (33, 34, 43).

This study enables pharmacist's perception regarding vaccination within a community pharmacy. One of the strengths of our analysis is represented by the complexity of practice settings and specializations of the participants who completed the survey. Specific barriers and benefits that could appear during vaccination services in community pharmacies have been addressed as the basis for governments to ensure the possibility to gain novel knowledge in European Faculties of Pharmacy.

The limitations of our study include the reduced number of respondents from all the selected countries, being hard to generalize the conclusions. Even more, the average age was rather low, therefore the data cannot be considered to represent elderly pharmacist colleagues. As the survey was conducted during the pandemic, many community pharmacists encountered more workload and worked under new conditions.

Our study could support pharmacist's professional work in public health systems and emergencies and could lead to future policy statements by providing recommendations from current health workers. Even more, WHO asked for collaboration between healthcare professionals. Our survey contributes to gathering data about pharmaceutical practice by reporting specific surveys and experiences. Although still there are legislative barriers and possible deficient reimbursements regarding vaccine administration in community pharmacies also confirmed by other studies (2, 3), targeted education programs among the Faculties of Pharmacy could further integrate pharmacist's efforts in reducing pandemic and natural disaster burden.

## Conclusions

Pharmacist's involvement in vaccination programs is proved as an effective practice for disease prevention and increasing the vaccination rate. Though many countries have implemented such programs as well as appropriate legislative requirements, still there are issues and barriers to the entire usage of pharmacist's knowledge, capacity, and capability in some South-East countries such as Albania, Bulgaria, Romania, and Serbia. Some types of resistance from pharmacists themselves to adopt these new roles to provide such services were demonstrated in Bulgaria, Romania, and Serbia, but not in Albania. The main limitation to the development of the pharmacist's role in vaccination was identified as being the limited access to training opportunities. Our study provides a piece of evidence to influence the pharmacy curricula revision and change. Vaccination training should be integrated into pharmacy education because it could enhance professional opportunities and could increase the recognition of community pharmacists as reliable and trustworthy healthcare professionals.

Also, for having a legal framework for pharmacists, it is necessary to have national regulations that define vaccination activities that pharmacists can operate, specific guidelines, and procedures for every activity that is related to vaccination in community pharmacies. Further policy and educational reforms considering national-based priorities and specifics are needed for a better understanding of and adopting the expanding role of community pharmacists.

## Data Availability Statement

The raw data supporting the conclusions of this article will be made available by the authors, without undue reservation.

## Ethics Statement

The studies involving human participants were reviewed and approved by the Ethics Commission of the University of Medicine and Pharmacy of Craiova, Romania. The patients/participants provided their written informed consent to participate in this study.

## Author Contributions

AT-S, MK, IT, and KH: conceptualization. AT-S and IT: methodology. MB and GP: validation. AT-S: formal analysis. M-SS and A-DM: investigation. SS, MO, JA, SS, M-SS, A-DM, and ET: data curation. AT-S, M-SS, and A-DM: writing and original draft preparation. MK, MB, IT, and KH: writing, reviewing, and editing. AT-S, MB, and GP: supervision. All the authors have read and agreed to the submitted version of the manuscript.

## Funding

The work of IT and MO was partially supported by the Ministry of Education, Science, and Technological Development of the Republic of Serbia (Project No. 451-03-9/2021-14/200161). This research received no external funding.

## Conflict of Interest

The authors declare that the research was conducted in the absence of any commercial or financial relationships that could be construed as a potential conflict of interest. The reviewer SD declared a shared affiliation with several of the authors, IT, SŠ, and MO, to the handling editor at the time of review.

## Publisher's Note

All claims expressed in this article are solely those of the authors and do not necessarily represent those of their affiliated organizations, or those of the publisher, the editors and the reviewers. Any product that may be evaluated in this article, or claim that may be made by its manufacturer, is not guaranteed or endorsed by the publisher.
